# Screening and management of dyslipidemia in oncologic patients undergoing cardiotoxic therapies: results from an Italian survey

**DOI:** 10.1186/s40959-023-00183-0

**Published:** 2023-08-05

**Authors:** Massimiliano Camilli, Irma Bisceglia, Maria Laura Canale, Fabio Maria Turazza, Leonardo De Luca, Domenico Gabrielli, Michele Massimo Gulizia, Fabrizio Oliva, Furio Colivicchi

**Affiliations:** 1 Department of Cardiovascular and Pulmonary Sciences, Catholic University of the Sacred Heart, Rome, Italy; 2 Department of Cardiovascular Medicine, Fondazione Policlinico Universitario A. Gemelli IRCCS, Rome, Italy; 3 Integrated Cardiology Services, Cardio-Thoracic-Vascular Department, Azienda Ospedaliera San Camillo Forlanini, Roma, Italy; 4 Cardiology Department, Nuovo Ospedale Versilia Lido Di Camaiore, Di Camaiore, LU Italy; 5 Cardiology Unit, Fondazione IRCCS Istituto Nazionale dei Tumori, Milan, Italy; 6 Cardiology Unit, Cardio-Thoracic-Vascular Department, Azienda Ospedaliera San Camillo Forlanini, Roma, Italy; 7 Cardiology Department, Azienda di Rilievo Nazionale e Alta Specializzazione “Garibaldi”, Catania, Italy; 8 Fondazione per il Tuo cuore-Heart Care Foundation, Firenze, Italy; 9 Cardio-Thoracic-Vascular Department “A. De Gasperis”, ASST Grande Ospedale Metropolitano Niguarda, Cardiology 1, Milan, Italy; 10 Clinical and Rehabilitation Cardiology Department, Presidio Ospedaliero San Filippo Neri, ASL Roma 1, Roma, Italy

**Keywords:** Dyslipidemia, cancer patients, National survey, Statins, Cardio-oncology

## Abstract

**Background:**

Baseline cardiovascular risk factors correction is recommended in all cancer patients undergoing potentially cardiotoxic therapies. Despite available guidelines, real-world data on dyslipidemia prevalence and management in the oncologic population are still sparse.

**Methods:**

This survey was an Italian, investigator-initiated survey initially designed and drafted by the Cardio-Oncology section of the Associazione Nazionale Medici Cardiologi Ospedalieri (ANMCO), comprising 10 individual multi-choice questions and spread after validation through the ANMCO mailing list. The survey was sent to cardiologists working in cardio-oncology units and/or managing patients with cancer.

**Results:**

Our survey included 139 Italian cardiologists. The majority of them routinely ask for the baseline lipidic profile of their patients, regardless of previous clinical history and planned treatment. According to our participants, the estimated prevalence of dyslipidemia in this population is between 20% and 60%. Although this high prevalence, our results highlight that there is poor harmony in terms of scores for CV risk prediction used in clinical practice to guide drug prescription and baseline therapy optimization. On the same line, coronary artery calcium score is poorly used in this setting. At the same time, more than 30% of interrogated physicians do not prescribe adequate statin doses, even though necessary, and have uncertainties on the use of other anti-dyslipidemic drugs in this population.

**Conclusions:**

Our results highlight the necessity of strong evidences on dyslipidemia screening and management in the cancer population, as well as the need of knowledge diffusion from scientific societies to clinicians treating these patients.

## Introduction

Reduction in left ventricular (LV) systolic function and occurrence of overt heart failure (HF) are well-known potential consequences of chemotherapy [1]. In particular, in the field of cardio-oncology, anthracyclines are the most studied compounds [1] causing cardiotoxicity, mainly through production of reactive oxygen species and topoisomerase II–mediated cell death [1]. Interest in the use of statins as a preventive strategy against anthracycline-mediated cardiotoxicity focuses on the pleotropic effects of these drugs. Of importance, in a recent randomized, placebo-controlled study, atorvastatin 40 mg intake revealed to significantly reduce left ventricular ejection fraction (LVEF) drop in 286 patients undergoing anthracyclines at a median dose of 300 mg/m^2^ for hematologic malignancies [2]. Nevertheless, in oncologic patients, myocardial dysfunction may be also caused by accelerated coronary artery disease (CAD), due to previous exposure to radiotherapy, underlying chronic inflammatory status and shared risk factors with cancer, including smoking habits, obesity and dyslipidemia [1].

Besides the growing body of evidence highlighting the need of treating hypercholesterolemia to mitigate CAD occurrence and of implementing cardioprotective strategies through the use of statins, real-word data on management of hypercholesterolemia in the cancer population are missing. We therefore designed a web-based survey asking about views and experiences on management of dyslipidemia in this highly-vulnerable population.

## Methods

This survey was an Italian, investigator-initiated survey initially designed and drafted by the Cardio-Oncology section of the Associazione Nazionale Medici Cardiologi Ospedalieri (ANMCO). The survey material consisted of 10 individual multi-choice questions and was spread after validation through the ANMCO mailing list. The survey was sent to cardiologists working in cardio-oncology units and/or routinely managing patients with cancer. Questions were available for 3 months (from 13 to 2022 to 12 March 2023) on the web platform with multiple invitations sent, reaching a total of 434 physicians. Categorical variables are expressed as percentages.

## Results

**Fig. 1:**
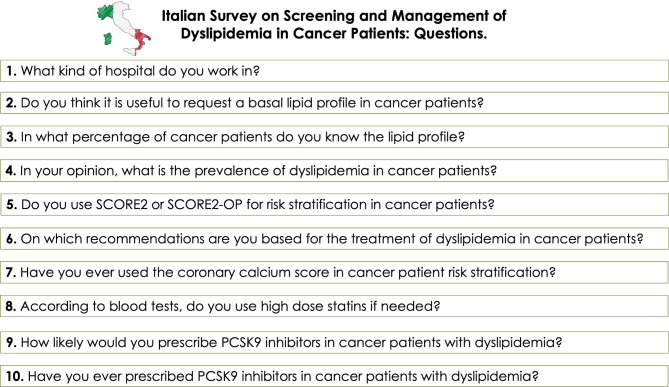
Questions proposed to the audience Legends. PCSK9: proprotein convertase subtilisin/kexin type 9

139 Italian cardiologists completed the survey and 100% of participants answered to all the questions. The vast majority of participants were practicing in general hospitals (61,9%), university or not, while 38,1% worked as ambulatory cardiologists. Questions presented are available in Fig. 1.

More than 70% of clinicians routinely asked for the baseline lipidic profile in cancer patients, regardless of previous clinical history and planned treatment. However, a not negligible proportion of participants investigated this aspect only if patients had a diagnosis of CAD (11,5%) or only if subjects underwent chemotherapy potentially inducing cardiac ischemia (11,5%). The majority of physicians (above 60%) reported to know the lipidic profile of more than 40% of their oncologic patients and basing on their experience, estimated the prevalence of dyslipidemia in this population between 20% and 60%. While almost all participants relied on European Society of Cardiology (ESC) recommendations on cardiovascular (CV) disease prevention, 56,1% of them did not routinely use the advised scores for CV risk prediction (e.g. SCORE2, SCORE2-OP) [3]. At baseline evaluation, only the minority of physicians asked to radiologists for coronary artery calcium (CAC) score quantification (2,9%) and used it to guide risk factors control.

Relating to management of dyslipidemia, 30,9% of clinicians did not use the recommended dose of statin because of possible pharmacological interactions with chemotherapy schemes, while 20,9% highlight the lack of evidences in this population. On the other hand, as regards the use of proprotein convertase subtilisin/kexin type 9 (PCSK9) inhibitors, 64% of physicians have never used these drugs in the oncologic population, despite the majority of them would be prone to prescribe this compound.

**Fig. 2:**
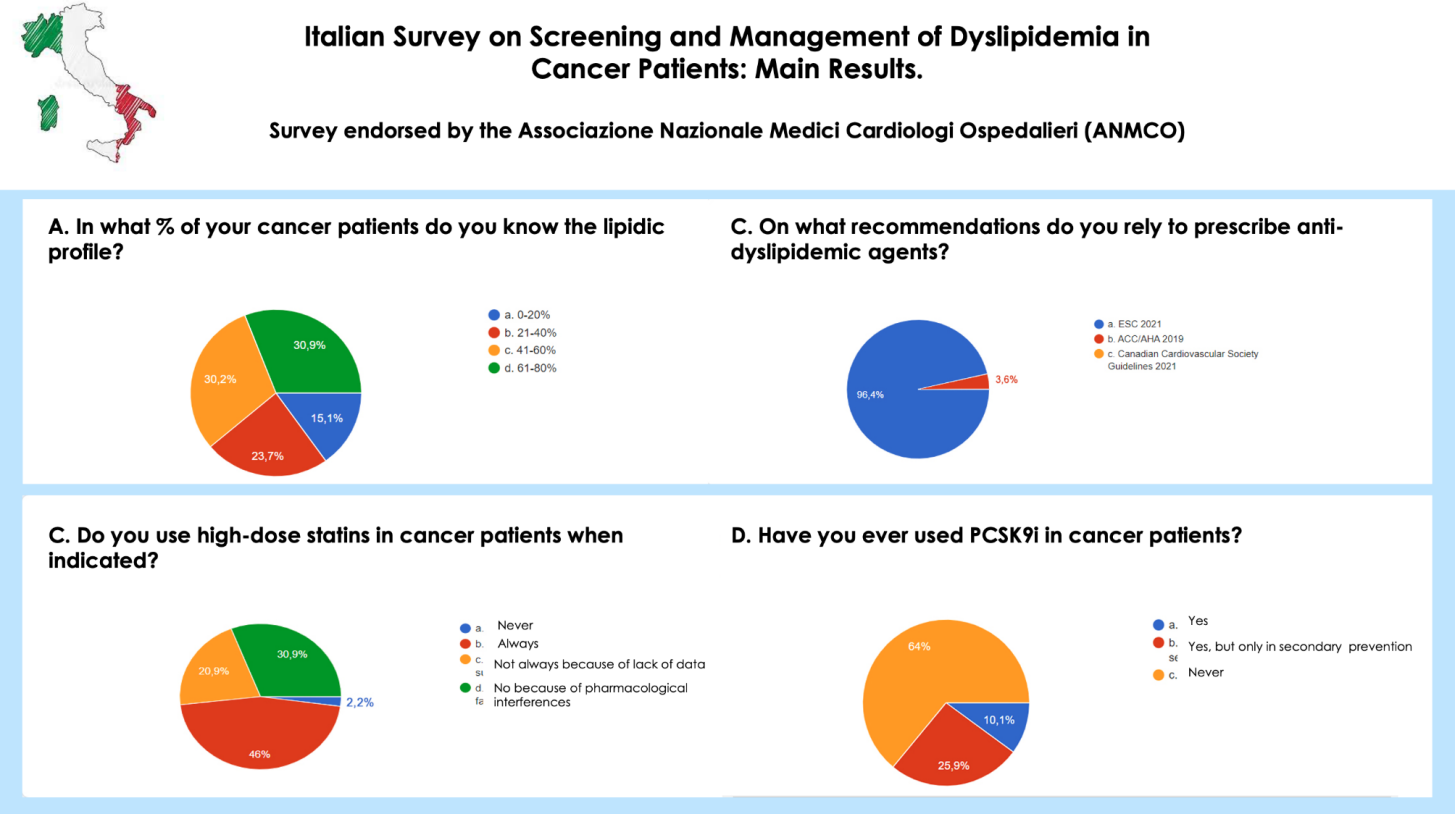
Main results of the survey on screening and management of dyslipidemia in cancer patients Legends. ACC/AHA: American College of Cardiology/America Heart Association; ESC: European Society of Cardiology; PCSK9i: proprotein convertase subtilisin/kexin type 9 inhibitors

Main results are presented in Fig. 2.

## Discussion

Hyperlipidemia is a common comorbidity among cancer patients and survivors, with significant differences in terms of lipid profiles among different types of malignancies [3, 4] and with deep impact on outcomes, enhancing cardiac and vascular toxicity of anticancer therapies.

This is the first survey investigating dyslipidemia prevalence and management in cancer patients undergoing potentially cardiotoxic treatment. Our main findings are: (i) when facing with an oncologic patient undergoing cardiotoxic treatment, the majority of participants screen for hypercholesterolemia; (ii) estimated prevalence of dyslipidemia in cancer patients is largely variable; (iii) clinicians do not routinely apply CV risk scores recommended by guidelines when dealing with oncologic patients; (iv) high statin doses, even if necessary, are rarely used because of the fear of potential pharmacological interferences; (v) most of participants consider feasible starting PCSK9 inhibitors in this subgroup of patients, but do not routinely prescribe these compounds.

For decades, CV complications of chemotherapy have represented an unsolved enigma for. cardiologists, resulting in CV undertreatment of the oncologic population. For this reason, dedicated cardio-oncology teams have been created, in order to improve patients’ outcomes, management and surveillance. The ESC Cardio-Oncology guidelines have been recently released [1]. While these recommendations advise CV risk factors assessment at baseline and frequent re-evaluation during and after chemotherapy, management of hypercholesterolemia is not adequately addressed. Moreover, hypercholesterolemia is not included in proformas used before chemotherapy to characterize patient’s risk of cardiotoxicity.

At the same time, clinicians rarely rely on traditional CV risk assessment tools used for the general population [5], which actually do not include cancer-specific parameters and may underestimate the true long-term CV risk in this population.

Furthermore, our data underscore that CAC score is rarely used as aid for risk stratification. This parameter is easily retrievable from computed tomography scans that patients undergo for tumor staging [6] and may guide primary prevention strategies.

Pharmacological interactions between chemotherapy compounds and CV medications are frequent and may represent a reason of undertreatment of cancer patients [7, 8]. In particular, many commonly-prescribed statins [7, 8] may have interferences with several regimens used in oncology, potentially causing changes in the disposition of medicines that could significantly alter their effect and cause toxicity. From our results, Italian cardiologists, despite routinely managing cancer patients, are not confident in prescribing the adequate doses of statins, even when necessary. Data about the effect of lipid-lowering compounds in cancer patients should be retrospectively collected from existing registries and hence, implemented in clinical practice. In addition, evidences on novel pharmacological therapies are lacking (e.g. PCSK9 inhibitors) and prospective studies should recruit patients with history of cancer.

Our paper has limitations. This is a national survey, only involving participants from Italy and belonging to the same medical association (ANMCO). Moreover, the knowledge in the field of cardio-oncology is still limited, which prevented wide participation to our survey. At the same time, the survey had an open access, and therefore, we cannot affirm that all the responders were effectively physicians. Lastly, many survey participants worked in tertiary care centers. Therefore, these findings may not be generalizable to all clinical environments and may need external validation.

## Conclusions and perspectives

Our results highlight the lack of strong evidences on dyslipidemia screening and management in the cancer population, as well as the need of knowledge diffusion by scientific societies directed to clinicians treating these patients. The aim of this paper is to grow awareness on this topic and to stimulate the creation of practical roadmaps, based on current knowledge gaps and tailored on the cancer patient, in which cardio-oncology units play a pivotal role. At the same time, properly-designed clinical studies are needed, starting from real-word observational data up to dedicated pharmacological trials, in which cancer patients will be at the center of the stage. Moreover, trained physicians are necessary for addressing both pharmacodynamic and pharmacokinetic interactions and for recommending dose adjustment or alternative therapies to ensure safe and effective medication use.

## Data Availability

The datasets used and analysed in this study are available from the corresponding author on reasonable request.

## Abbreviations

ANMCO: Associazione Nazionale Medici Cardiologi Ospedalieri
CAC: Coronary artery calcium
CAD: Coronary artery disease
CV: Cardiovascular
ESC: European Society of Cardiology
LVEF: Left ventricular ejection fraction
PCSK9: Proprotein convertase subtilisin/kexin type 9
